# Supplementary material for: Fitness landscape of a dynamic RNA structure
Source: PLoS Genet. 2021 Feb 1;17(2):e1009353. doi: 10.1371/journal.pgen.1009353 (PMC7877785; doi:10.1371/journal.pgen.1009353)
Supplement: S2 Table — (DOCX) [file pgen.1009353.s010.docx]

**S2 Table. XGBoost models**

| **Measure predicted** | **Features** | **Temperature** | **Genotypes included** | **Spearman’s correlation (predicted v observed)** |
| --- | --- | --- | --- | --- |
| Fold-change (DESeq2) | Nucleotide identities only | 30ºC | all | 0.84 |
| Fold-change (DESeq2) | Nucleotide identities only | 37ºC | all | 0.83 |
| Fold-change (DESeq2) | Nucleotide identities only | 30ºC | P_adj_<0.05 | 0.77 |
| Fold-change (DESeq2) | Nucleotide identities only | 37ºC | P_adj_<0.05 | 0.74 |
| Fold-change (DESeq2) | Nucleotide identities only | 30ºC | P_adj_<0.01 | 0.75 |
| Fold-change (DESeq2) | Nucleotide identities only | 37ºC | P_adj_<0.01 | 0.71 |
| Fold-change (DESeq2) | Extended* | 30ºC | P_adj_<0.05 | 0.77 |
| Fold-change (DESeq2) | Extended* | 37ºC | P_adj_<0.05 | 0.74 |
| Fold-change (DESeq2) | Extended* | 30ºC | P_adj_<0.01 | 0.76 |
| Fold-change (DESeq2) | Extended* | 37ºC | P_adj_<0.01 | 0.71 |
| Wald statistic (DESeq2) | Nucleotide identities only | 30ºC | all | 0.81 |
| Wald statistic (DESeq2) | Nucleotide identities only | 37ºC | all | 0.80 |

*extended runs include the following features together with nucleotide identities: RNAfold-predicted ensemble free energy, RNAfold-predicted ensemble diversity, number of possible strong (G-C) on-target base-pairs in P1ex, number of possible weak (A-U) on-target base-pairs in P1ex, number of possible wobble (G-U) on-target base-pairs in P1ex, number of opposing bases that would not form a base-pair in P1ex, base-pairing status of N_2_-N_21_ (strong, weak, wobble, none), base-pairing status of N_3_-N_20_, base-pairing status of N_4_-N_19,_ base-pairing status of N_5_-N_18_, number of possible strong (G-C) on-target base-pairs in P10, number of possible weak (A-U) on-target base-pairs in P10, number of possible wobble (G-U) on-target base-pairs in P10, number of opposing bases that would not form a base-pair in P10, base-pairing status of N_20_ with P10, base-pairing status of N_19_ with P10, base-pairing status of N_18_ with P10, Hamming distance, GC content of the N_2_-N_5_/N_18_-N_21_ genotype.
