# Supplementary material for: Fitness landscape of a dynamic RNA structure
Source: PLoS Genet. 2021 Feb 1;17(2):e1009353. doi: 10.1371/journal.pgen.1009353 (PMC7877785; doi:10.1371/journal.pgen.1009353)
Supplement: S3 Table — (DOCX) [file pgen.1009353.s011.docx]

**S3 Table.** Oligonucleotides used in this study. “f” indicates a forward primer and “r” indicates a reverse primer. Mixed bases are in blue. The restriction sites are underlined. The Illumina adapter sequences are boxed.

| **Oligonucleotide** | **Sequence (5’ → 3’)** |
| --- | --- |
|  | |
| **Subcloning of the *knt-intron* sequence into pET-22b(+) plasmid** | |
| knt-rz-f | GTGTGAGGCATATGAATGGACC |
| knt-rz-r | TAGAGGTCTCGAGTTAAAATGGTATGCGTTTTGAC |
|  |  |
| **Generation of mutagenic Fragment 1 (Figure S7)** | |
| Frag1-f | GATCAGCCCACTGACGCGTTGC |
| Frag1-r | CGTAATATTGCTNNNNAGAGAGCCATAAACACCAATAG |
|  |  |
| **Generation of mutagenic Fragment 2 (Figure S7)** | |
| Frag2-f | AGCAATATTACGNNNNGGAGGGAAAAGTTATCAGGC |
| Frag2-r | ACCTGAGATGCATAATCTAGTAGAATCTC |
|  |  |
| **Amplification of P1ex sequences from plasmid pools** | |
| C20Aseq-f | TCGTCGGCAGCGTCAGATGTGTATAAGAGACAGGGGGATGATGTTAAGGCTATTGGTGTTTATGG |
| C20Aseq-r | GTCTCGTGGGCTCGGAGATGTGTATAAGAGACAGCGGTCTTGCCTTTTAAACCGATGCAATC |
|  |  |
