# Supplementary material for: Fitness landscape of a dynamic RNA structure
Source: PLoS Genet. 2021 Feb 1;17(2):e1009353. doi: 10.1371/journal.pgen.1009353 (PMC7877785; doi:10.1371/journal.pgen.1009353)
Supplement: Attachment — Submitted filename: 108996_1_rebuttal_1877083_qhzzl0.pdf [file pgen.1009353.s013.pdf]

Point-by-point response to reviewers

Reviewer #1 (Evidence, reproducibility and clarity (Required)):

1. *The authors constructed a virtually complete fitness landscape of the P1 extension region (4-base-paired helix) in the group I intron from Tetrahymena thermophila, using a kanamycin resistance reporter to evaluate the fold-change in fitness, which is related to self-splicing activity. This was a clever choice of system because it was known from earlier work that the P1 extension adopts two different conformations during self-splicing. The fitness of each variant was determined from the number of reads acquired from the sequencing data sets and analyzed through an extensive computational pipeline.*

   The strength of the paper is that this machine learning approach can be used to calculate how individual variants contribute to the fitness landscape and assess the directions of epistasis across a large number of identified genotypes.

We thank the reviewer for highlighting one of the key strengths of our manuscript, the fact that our analytical approach, using SHAP values, enables contributions of individual variants to be assessed in a genotype-specific manner. This approach provides for a sound, robust, and principled way of describing and understanding the fitness impact of one mutation in the context of (potentially many) others.

2. *The authors argue that machine learning more successfully models subtle effects that arise from interactions between RNA residues, and that the power to analyze deep mutational sequencing experiments can better rationalize fitness constraints arising from multiple conformational states.*

We do indeed argue that machine learning is likely to play an increasing role in making sense of deep mutational scanning data. These scans provide high-resolution information on how fitness maps onto genotype, but the molecular underpinnings of this relationship often remain obscure. It is these “hidden” underpinnings, including the effects of specific mutations on RNA/protein folding, structures, and dynamics, that machine learning approaches can help elucidate.

3. *The results are mostly consistent with previous studies even though the authors collected the data in a more advanced and complicated way. They are also able to rationalize complex phenotypes - for example, the observed fitness defects are more prevalent under an unfavorable growth condition (30ºC), because the lower temperature hinders conformational exchange. Although such cold sensitive effects are well known in RNA, it is gratifying that this can be captured in the fitness landscape.*

Finding temperature-related fitness effects that are consistent with impaired conformational exchange was also gratifying for us and we thank the reviewer for highlighting this finding.

4. *The results would be more convincing if the authors directly measure the self-splicing activity of a few key variants, such as the C2C21 mutant, to determine whether these mutations alter the self-splicing mechanism of the Tte-119(C20A) master sequence in the way that they infer from their model. In interpreting their results, they may want to consider misfolding of the intron core (coupled to base pairing of P1) and reverse self-splicing. Reversibility in the hairpin ribozyme, for example, turned out to be the key for understanding the effects of certain mutations.*

We appreciate that measurements of splicing activity for individual genotypes would complement and further strengthen our study. We will therefore aim to construct strains for a few key genotypes and assay self-splicing activity using RT-qPCR – an approach we previously used successfully to monitor splicing kinetics of self-splicing introns in yeast mitochondria (see Rudan *et al*. 2018 eLife 7:e35330). Specifically, we will quantify the fraction of spliced and unspliced transcripts using primers that span the exon-exon and the 3' exon-intron junction, respectively (the 5' intron-exon junction is genotypically diverse and would require genotype-specific primers). This will be done under non-selective (-kan) conditions, where the relative fraction of spliced and unspliced transcripts is a function of intrinsic splicing ability and not confounded by selection. We aim to include the master sequence, C2C21, G3C20 and its mirror genotype C3G20, U3 (which restores perfect complementarity in the master sequence), and G5 (inferred from the high-throughput experiment to make a strong negative contribution to fitness).

In interpreting our results, we will consider different mechanisms of splicing failure, such as kinetic problems (slow dissociation of P1ex), misfolding of the intron core, reverse self-splicing, and the use of cryptic splice sites, which has previously been documented (see e.g. Woodson & Cech 1991 Biochemistry 30:2042-2050). We note, however that a precise mechanistic dissection of the splicing defects of individual variants is not the purpose of this manuscript and we therefore do not aim to establish genotype-specific defects in great molecular detail.

5. *Related to the point above, interesting conclusions regarding the relationships between base identity and epistasis that arise from metastability should be strengthened with additional examples. For example, the authors can explain why a reverse base-pairing variant (C3G20) exhibits negative epistasis but is not similar to that of the G3C20 construct. This would ideally use the data from the screen but also be validated by checking the self-splicing activity of a few individuals at low and high temperature.*

In measuring splicing activity and its link to fitness for a subset of key variants (see point #4), we will include at least one mirror example such as C3G20/G3C20. In addition, we will highlight additional examples of this mirror asymmetry based on the results from our high-throughput screen.

6. *They should validate the screen by showing that kanamycin resistance does indeed correlate strictly with self-splicing activity, and not some other feature such as RNA turnover. (It would also not be a bad idea to check this in the cell, which can be done by primer extension or Northern blotting.)*

This question (i.e. whether altered RNA stability rather than splicing efficiency explains differential KNT production and ultimately fitness) has previously been addressed by Guo & Cech (2002) when introducing the *knt*+intron reporter system. These authors found no difference in mRNA stability in constructs that displayed differential kanamycin resistance. To shore up this conclusion further, we will measure fitness (via colony counts, growth rate or more directly through competitive fitness assays) of the key variants for which we determine splicing activity (see point #4) and then correlate splicing and fitness.

7. *The benefit of the machine learning model is that it can extract signals that may be hard to detect otherwise. The downside is that it doesn't produce a physical model, as far as I am aware. The parameters are themselves not meaningful - except to the degree that trends in the fitness estimates can be explained after the fact. This is something that should ideally be explained more directly in the manuscript.*

The reviewer raises an interesting point, that indeed deserves further discussion/explanation. The reviewer is right that, at first sight, high-resolution fitness landscapes like ours do not directly produce a physical (structural) model of the molecule under investigation. They connect genotype and fitness, but the molecular intermediate – a biophysical structure – is not explicit. However, over the last few years, it has become apparent that deep mutational scanning experiments can – both in principle and in practice – yield information that can be leveraged to infer such a physical model. In short, covariation in fitness between residues in a protein or bases in an RNA can be used as inputs for constraint-based modelling of physical interactions. Notably, Schmiedel & Lehner (2019, Nature Genetics 51: 1177-1186) recently demonstrated that deep mutational scanning data can be used in this manner to reconstruct secondary and tertiary protein structure with high accuracy. In principle, the same approach can be used to reconstruct RNA structures. This will require more extensive, molecule-wide fitness data, but our study points towards just this future, even for data collected from structural ensembles.

When we stated in the original manuscript that deconvolution of the fitness landscape might help to reverse engineer structures, this ability to interpolate between genotype and fitness to reveal hidden biophysical/structural relationships is what we refer to. We will revise the manuscript to make this connection more explicit.

8. *The authors claim that by evaluating a large number of sequences at two conditions, they can capture variants with intermediate phenotypes (Fig. 1). This is not necessarily true. If the original screen allows only the most active variants to survive on kan+ medium, then the signature of intermediate phenotypes may not be encoded in the original data, and thus not retrievable even with sophisticated algorithms, which may also be prone to overfitting. At what limit of stringency will the screen fail to yield information about intermediate fitness? How deeply must one sequence to recover this information, especially if noisy or degraded? Some discussion of these effects would be helpful.*

The capacity of any high-throughput sequencing-based DMS experiment to resolve intermediate phenotypes does indeed depend on a number of things. The reviewer highlights two of these: First, in screens where the phenotype is not binary (dead/alive) but fitness can be measured on a continuous scale, can we – and do we – capture phenotypes with

intermediate fitness? What if only the fittest/most active variants survive? This is, ultimately, an empirical question, and one we can answer quite definitively: we *do* observe a large range of intermediate phenotypes, which – in our study – correspond to intermediate fold-change values. For each genotype, we can provide confidence limits and assess statistical significance. Table S1 provides this information. Our capacity to resolve these intermediate phenotypes is mainly based on three things. One is adequate sequencing depth, as highlighted by the reviewer. The second is the number of biological replicates (N=6) we analyse, which allows us to differentiate biological variability from noise for a large number of genotypes. This is an important aspect of DMS experiments that has often been overlooked (i.e. there are many other studies where only a single replicate is analysed and biological heterogeneity is not taken into account). With six replicates in hand, we can directly estimate variability (as done e.g. in our DESeq2 analysis) and quantify uncertainty so as to guard against overfitting. In our view, this is arguably more important than sequencing depth in deriving appropriate fitness estimates. Finally, we can resolve intermediate phenotypes because we keep the time lag between initial exposure to kanamycin and assaying genotype frequencies relatively short (overnight growth, see Methods). Our experiment is effectively a multi-genotype competition experiment, and we provide a snapshot across the genotype pool at a given time. If we had measured after several days of culture, genotypes with greater relative fitness would have spread further through the population, at the cost of less fit genotypes, many of which would likely have been eliminated. We kept measurement lag relatively short on purpose so that we could see a clear differential response to kanamycin while still being able to catch more than just a handful of the very fittest genotypes.

In light of the above, it will be apparent that there are no simple answers to the reviewer's questions about required sequencing depth, levels of stringency, etc. The ability to assign differential fitness across a large population of genotypes hinges on multiple interrelated considerations (sequencing depth, complexity of the final & starting pool, number of replicates). In revising the manuscript, we will highlight some of the key considerations just discussed, bearing in mind that the manuscript cannot possibly discuss all possible pitfalls and requirements of deep mutational scanning experiments in great detail.

9. *Lastly, the evolvability of RNA is fascinating and there is much to learn. However, the authors don't discuss the implications of their findings for molecular evolution although they throw the term around. It would be exciting if there is a trend in the fitness landscape that could help explain the trajectory of RNA evolution in nature.*

We agree with the reviewer that it would be exciting to link deep mutational scanning results more closely with observable patterns of RNA evolution. This is true both in relation to evolution of P1ex/group I introns specifically and evolution of dynamic RNA structures more generally. Regarding the latter, we note that selection against excess stability has previously been inferred for 5' UTRs (see e.g. Gu *et al.* 2010 PLoS Comp Biol 6: e1000664), although our case is slightly different in that a helix still needs to form but be sufficiently unstable to enable swift dissociation. We also note that riboswitches might make for an excellent subject to study asymmetric constraint and selection against excess stability as they involve formation of competing helices (including participation of some but not all nucleotides in more than one helix), their structure/function is well understood, and many examples are known, providing opportunities for evolutionary analysis. We consider this outside the scope of the current study. We will, however, seek to analyse patterns of evolution in P1ex to

establish whether they correspond in a meaningful way to the fitness trends we observe in the laboratory. To do so, we will analyse the distribution and evolutionary history of variants across orthologous introns in different Tetrahymena species/strains, with a focus on P1ex, P10 and the surrounding sequence. Fortunately for us, the 23S ribosomal RNA gene in which the intron is embedded has been used as a phylogenetic marker so that intron/exon sequence information is available for a reasonable number of species/strains (see Doerder 2018 J Eukaryot Microbiol 66:182-208). We will generate an alignment of these sequences and ask, for example, whether N_2-N_5 are subject to different constraints than N_{18}-N_{21} mirroring our experimental findings. We have previously successfully quantified patterns of variation surrounding self-splicing introns in yeast mitochondria (Repar & Warnecke 2017 Genetics 205:1641-1648). Note here that extending this analysis beyond Tetrahymena is problematic. Specifically, the intron is absent from close relatives of Tetrahymena (Doerder 2018 J Eukaryot Microbiol 66:182-208) and P1-proximal structures of distant relatives are quite variable. In addition, we are looking at intronic regions that are not only adjacent to but also directly interact with exonic sequence. The exonic context in which the intron is embedded therefore matters but will be quite different for more distant group I introns. We therefore think that aligning and comparing distant orthologs has limited merit.

10. *The authors use the abbreviation DMS for deep mutational scanning; the RNA structure field uses the reagent dimethylsulfate that is also abbreviated DMS. They may want to choose a different acronym or just avoid an acronym altogether.*

We appreciate this point about false-friend acronyms. We will either find a different acronym or avoid it altogether.

Reviewer #1 (Significance (Required)):

11. *As the importance of RNA structure for gene expression becomes more widely appreciated, interest in understanding the evolution of RNA structures is also increasing. Compared with the molecular evolution of proteins, evolution and fitness in RNA is far less understood, although the authors appropriately point to a number of recent studies on this topic. The main advance here is to use machine learning methods to analyze the results of a large genotypic screen, with the goal of more accurately capturing the fitness effects of sequences at varied distances from the parental sequence. The specific conclusions reached here such as the importance of metastability or the prominence of cold sensitive effects are not revolutionary, but the authors illustrate how such phenomena can be investigated more systematically and in more depth.*

We thank the reviewer for highlighting that our analytical approach showcases how deep mutational scanning data can be analysed in an unbiased and systematic manner to better understand the relationship between genotype, molecular phenotype (e.g. structure), and fitness. The reviewer also rightly points to specific results we obtain regarding temperature-related effects and metastability of P1ex/P10. However, we believe that the most important contribution of this work is a more general one, namely our proof-of-principle demonstration that deep mutational scanning data can capture multiple conformational states simultaneously, and that these states can be deconvoluted from a single fitness landscape to attribute the fitness impact of individual mutations to specific RNA conformations. To our

knowledge this had not been explicitly demonstrated before and our work provides an important cornerstone for future studies looking to interpret mutational effects in either RNAs or proteins in the light of dynamic structures.

In light of comments by reviewer #2 below, it is worth reiterating the proof-of-principle nature of this study. Many of the specific results we obtain (e.g. importance of avoiding excess stability in P1ex) are not revolutionary. Indeed, we would be worried if they were. We chose to investigate P1ex because substantial prior work exists that has furnished us with solid positive controls. This independent prior validation allows us to both have great confidence in the data we generate and demonstrate cogently that the two conformational states at the beginning and end of the splicing reaction are captured in the data.

Finally, we believe our work, in covering a virtually complete genotype space, using multiple replicates to quantify uncertainty in fitness estimates, and using SHAP scores to interpret variant effects in genotype-specific context, sets a new high bar for this type of study and will provide valuable reference data and analytical recipes for future analyses.

Reviewer #2 (Evidence, reproducibility and clarity (Required)):

12. *The manuscript by Soo et al probes the effect of mutations on the fitness of the Tetrahymena Group I self-splicing intron. They used high-throughput sequencing to simultaneously identify the effect of every possible sequence in a 4-bp helix. The approach is sound and the conclusions are generally supported. However, the analysis seems overly complicated given the dataset. Both the analysis and the accompanying writing make it difficult to understand what seems to be a fairly clear conclusion - that the relative stabilities of two alternative RNA helices are important for splicing.*

We thank the reviewer for testifying to the validity of our approach and the soundness of our conclusions. Regarding the complexity of the analysis, the reviewer is right in that – for the conclusion that the relative stabilities of two alternative helices are important for fitness – a simpler analysis would have sufficed. However, as elaborated in response to point #11 above, our objective here is not merely to draw specific conclusions about the relative stabilities of P1ex and P10, but more general: a) to demonstrate that a single fitness landscape can be deconvoluted to implicate multiple conformations in fitness defects and b) to provide a basic but powerful recipe for doing so in an unbiased, systematic manner using machine learning. We will strive to make the writing clearer so that readers can follow this reasoning and appreciate our analytical choices.

Major Comments

13. *The authors state that this method can identify the impact of transient conformational states. However, the two conformational states in this study are not transient - in fact they are associated with two distinct chemical steps of splicing and are quite stable. It may be that the effect of important transient states would be observed, but this study does not demonstrate that.*

We used the word “transient” to describe two alternative RNA structures formed during the life cycle of the intron. Both states (characterized by P1ex and P10 formation) are transient in as much as they disappear as splicing proceeds. In retrospect, we agree with the reviewer that this usage is too loose (given how the term is generally used in the literature) and might evoke the wrong connotations. We will therefore revise the manuscript to eliminate references to P1ex and P10 as transient states, but rather describe them as alternative conformations. Of course, the general point remains true: that deep mutational scanning data should in principle capture all fitness-relevant structural states even if these are transient (in the strict sense of the word).

14. *"Fitness" ends up being on an arbitrary scale, which impairs some analysis. A similar high-throughput sequencing pipeline could have been used to directly monitor splicing of every mutant, though at this point that is outside the scope of this study. Even with the arbitrary units, it would be clearer if more time were spent comparing fitness to base-pair stability on an individual basis, rather than the broad analyses. (See minor comments for details.)*

The reviewer is right in saying that a high-throughput pipeline could have been designed to monitor splicing of each genotype directly (rather than assaying fitness of the cell population that represents a particular genotype).We chose not to do so. One reason for this is that monitoring splicing directly would have necessitated design of a more complicated assay. This is because, to monitor splicing efficiency, one would have to monitor both pre-mRNA and mRNA for different genotypes. The former is straightforward (using primers that span the exon-intron junction) but the latter is not: successful splicing removes the genotype-specific information from the mRNA (that information being solely encoded in the intron). This a solvable problem in principle. One might, for example, introduce barcodes of sufficient complexity in the mRNA that can be linked back to the intron genotype, but doing so would have introduced a further source of error and complicated analysis. We therefore opted for monitoring genotypic fitness by sequencing the plasmids from which the RNAs originate. This does mean that our measurements of fitness are not coupled to a specific molecular phenotype (such as splicing efficiency) – we presume (but are not entirely sure) this is what the reviewer refers to when talking about fitness being on an “arbitrary scale”. However, fitness derived in this manner has the advantage of providing information that does not start from a mechanistic preconception. We ask how variant affects survival and reproduction of the cell without presuming specific mechanism and the results can therefore capture any mechanism, including those that we did not consider initially. The challenge then becomes to tease out possibly multiple mechanisms from unbiased data.

We will tackle the reviewer’s final comment, regarding analysis of base-pair stability, below in response to one of the minor comments (point #20).

Minor Comments

15. *The sentence in the abstract beginning "Using an in vivo report system..." is very difficult to comprehend. This is due both to the length of the sentence and the word usage. The final sentence of the abstract is similarly difficult. In general, the writing overemphasizes complexity at the cost of clarity.*

We will revise the entire manuscript to make the writing both clearer and more concise.

16. *Analysis of results in terms of "epistasis" obscures what could be a straightforward observation. This is the same as saying that mutants are not independent, or that their energetic costs are not additive. This follows obviously from the observation that the nucleotides being mutated are base-paired.*

Making explicit reference to "epistasis" is a considered choice. Framing results in terms of epistasis might be less familiar to readers grounded in RNA or protein biophysics/biochemistry, but is very much at the heart of thinking about the genotype-phenotype relationship from an evolutionary perspective, where global descriptions of epistasis are commonplace and usually provide the starting point for thinking about genotype-phenotype relationships, evolution and evolvability. So what seems unnecessarily obscure when seen through the lens of one field, is natural when considered in the context of another. Importantly, it is also the central approach adopted by many if not most prior deep mutational scanning studies (see e.g. Hayden et al. 2011; Pressman et al. 2019; Zhang et al. 2009; Li et al. 2016; Puchta et al. 2016; Domingo et al. 2018; Li and Zhang 2018; Weinreich et al. 2013; Lalić and Elena 2015; Bendixsen et al. 2017 as cited on page 3) so we think this framing is helpful to compare our results to prior work.

We expect that the readership will include many researchers interest in mapping genotype-phenotype-fitness relationships who will expect to see global analyses and descriptors of the type we present. We will, however, revise the manuscript to ensure that our description of the findings remains accessible to readers from other fields.

More specifically, we also note that the fact that mutations are not independent (i.e. epistasis exists) might be trivial from the fact that P1ex is a base-paired helix. The magnitude and direction ("sign") of epistasis, however, are not. In fact, as we describe, contrary to prior DMS on RNA helices, we find a lot of positive epistasis, reflecting, as we argue, selection against excess stability of P1ex to allow subsequent formation of P10.

17. *The novel information is the sensitivity of fitness to base pairing. This is best shown in an analysis like Figure 3A (see below), not broad measures of epistasis.*

Please see responses to points #11, #12, and #16 above for an elaboration of what we consider to be the main merits of this study and why providing broad measures of epistasis is a sensible choice.

18. *Figure 1C isn't necessary for the reader to understand the process.*

We are happy to follow editorial guidance as to whether this panel is superfluous and should be removed or is worth including.

19. *It is unclear what figure 2C is showing. It appears that the replicates are similar to each other, that 30 deg C and 37 deg C are also similar, but that +/- Kan are different. This probably doesn't need a figure in the main text.*

This figure does indeed capture what the reviewer describes: genotype pools in +/-kan are least similar to each other, while 30/37ºC are similar but distinct in the +kan condition and effectively indistinguishable in the -kan condition, in line with expectations. We agree with the reviewer that this information per se is something that would typically be found in a supplementary figure. However, we would advocate for retention of this panel in the main manuscript in this instance because of the way in which it was derived: using the Bray-Curtis dissimilarity index. To our knowledge, this is the first time that Bray-Curtis dissimilarity has been used to quantify, in a principled way, the similarity between genotype pools. Borrowed from the ecology literature, the index captures both richness (number of different species/genotypes in the ecosystem/genotype pool) and relative abundance to provide an integrated measure of genotype diversity. We believe that this measure will be useful for future studies and rather than relegating the figure to the supplement, we would aim to briefly highlight its methodological novelty.

20. *Figure 3A could be the most informative part of the manuscript. However, predicted minimum free energy should be on the x-axis as the independent variable. The expectation then is that you would see a peak in fitness at some free energy, with fitness falling off both with increased and decreased stability. Furthermore, there should be more analysis along these lines. The authors should calculate helical stability for both P1ex and P10 for every mutant and compare with fitness. Mutations which affect both could also be separated out. Figure 4C comes the closest to this but views it only in terms of GC pairs; there is no reason not to quantify the energetic effects given that predictions of stability for helices is quite good. Deviations from a model invoking only helical stabilities would indicate another factor is involved (alternative base-pairing or tertiary structure, for example).*

We agree with the reviewer that the axes in Figure 3A should be flipped and we will do so in the revised manuscript. We also agree that, when it comes to helical stability of P1ex, the simple expectation would be to see a peak at a certain stability with drop-offs either side, as intimated by Figure 4C. We further agree with the reviewer that Figure 4C is rather indirect and can be made more quantitative by considering helical stability across all genotypes directly. To this end, we will use one of the many tools available that allow prediction of helical stability from primary sequence (e.g. the enf2 function in RNAStructure, as used by Torgerson et al 2018 RNA, see point #24 below) and replace Figure 4C with a more quantitative fitness landscape based on these computations. To provide added confidence in the computations of helical stabilities from primary sequence in the context of our structure, we will also calculate helical stabilities from molecular dynamics simulations for the subset of genotypes we considered previously (Figure 4E/F) and see how inferred stabilities compare.

21. *There appears to be a missing verb in the legend for figure 3A, second sentence.*

We will fix this error.

22. *Figure S5 appears to be redundant with Figure 1.*

At first glance, Figure S5 does indeed appear redundant with Figure 1 but it is not. Figure S5 shows the relevant sequence of the group I intron and bordering exons in its native context, i.e. when embedded in the 23S ribosomal RNA gene of *Tetrahymena thermophila*, whereas Figure 1 shows the genotype of the mutant intron embedded in *knt*. The sequences are different. We will revise the legend to Figure S5 to make this clearer.

23. *Figure S6 is a better analysis than what appears in the main text, and could be expanded to all base pairs.*

We will expand Figure S6 to include all base pairs as suggested. We disagree that this is a better analysis compared to what appears in the main text. Rather, it provides a complementary, hypothesis-driven view whereas the analysis in the main text is more systematic and unbiased in approach.

Reviewer #2 (Significance (Required)):

24. *This manuscript largely focuses on the technical approach. The shift in analytic strategy described above would increase the conceptual impact. The conclusions are consistent with and fit in with recent uses of high-throughput sequencing to study RNA systems. For example Pitt & Ferré-D'Amaré, Science (2010) and Kobari et al, NAR (2015) describe fitness landscapes of the ligase and HDV ribozymes, respectively. Torgerson et al RNA (2018) make similar measurements on the glycine riboswitch, including a treatment of relative helix stability for two mutually exclusive conformations. The overall results are of interest to researchers in the field of noncoding RNA.*

We thank the reviewer for highlighting the paper by Torgerson et al, of which – embarrassingly – we were not aware. We will make reference to this paper in a revised manuscript and highlight that riboswitches might be a good model system to further explore asymmetric constraint and selection against excess stability in an evolutionary context (also see our response to point #9 above).

As highlighted earlier, we think the main conceptual impact of our work lies not in the description of helical stabilities. Rather, it lies in a) providing a rigorous proof-of-principle that deep mutational scanning can capture multiple conformational states simultaneously, and b) that, using an unbiased machine learning approach, these states can be deconvoluted from a single fitness landscape to attribute the fitness impact of individual mutations to specific RNA conformations. A shift in analytical strategy to "cut to the chase" and narrowly focus on helical stability would be misguided in this context, as we seek to provide not only insights into the data at hand but also lay out a sound and general recipe for analysing similar datasets in the future.
