# Supplementary material for: Fitness landscape of a dynamic RNA structure
Source: PLoS Genet. 2021 Feb 1;17(2):e1009353. doi: 10.1371/journal.pgen.1009353 (PMC7877785; doi:10.1371/journal.pgen.1009353)
Supplement: Attachment — Submitted filename: Review Comments Soo et al PlosGenetics 2020.docx [file pgen.1009353.s014.docx]

Review

Fitness Landscapes of a dynamic RNA structure

Nov. 18, 2020

The authors present data on the mutational effects in a region of a ribozyme sequence that is involved in structural changes important for function. Specifically, the authors mutate 8 nucleotides that are involved in both the P1ex and P10 elements of the Tetrehymena group I self-splicing intron, and several of the nucleotides must break and reform base-pairs to switch between the two structural elements during self-splicing. To study the combinatorial mutational effects, they clone their ribozyme library within a Kan resistance gene in a plasmid and transform E. coli with this plasmid such that accurate and efficient self-splicing alters growth rate. The ability to study fitness effects in the context of conformational changes is an important question for RNA biology and evolution, yet remains poorly understood. The manuscript presents interesting data towards a better understanding of this problem.

Major

The epistasis was calculated different than in Bendixsen et al 2017. This prior publication used a ratio of the products, not the difference. The equation in the legend of Figure 4 from this prior publication is *ε* = log_10_ (*W* _AB_ × *W* _wt_/*W* _A_ × *W* _B_)

Please resolve this difference and confirm that it does not change any conclusions. This may affect interpretation of asymmetrical fitness effects, for example.

The data shows that the sum of gains from machine learning is significantly higher for two-structure nucleotides (N18-N21) than single-structure nucleotides (N2-N5). However, it is not clear whether or not this can be used to identify alternative structures in other data sets, especially if the alternative structure is not previously known. If you take the distribution of all gains, can the N18-N21 be picked out without prior knowledge? If yes, this should be made more clear. If not, it should be discussed in terms of what is needed to be able to do this. I would think that catalytic and substrate binding nucleotides would swamp alternative structure data? Is there any evidence to the contrary?

Minor

The conclusions are based on a rather small data set. Comparing four nucleotides involved in two structures (N18-N21) to four nucleotides involved in one structure (N2-N5) is appropriate, but it is not clear how generalizable these results will be. I would prefer a more thorough discussion of the limitations of this data set, and guidance for future experiments or meta-analysis. Are there potentially other reasons for positively skewed epistasis and large proportion of gains that would not involve structural pleiotropy?

Another major conclusion in the paper is that their data is in support of the previous finding that an overly stable P1ex helix is unfavorable in terms of self-splicing. This is largely from interpretation of the difference in fitness effects at 30C and 37C. But, I do not find the interpretation of this data entirely convincing. The fitness effects in Fig. 2A show that the 30C data is more spread out in both positive and negative fitness values compared to 37C. The very low fitness mode is slightly more negative at 30C, but there are also more high fitness variants. I would trust the near-neutral fitness values over the very low fitness values. Importantly, I question the ability to distinguish differences between the lowest fitness values. When distinguishing between fitness ratio of 0.01 and 0.03, how many observations are even in the data? If these fitness effects are from a low number of observations, then they would be very sensitive to stochastic fluctuations that should not be over interpreted. Does the statistical analysis imbedded in the analysis pipelines allow a significant distinction between two low fitness sequences? This is different than a determination that the change in fitness is significant +/- antibiotic. As the authors point out, the stability has a very low correlation with fitness at 30C, and therefore has very limited predictive power. Why was this analysis not also done at 37C? The positive correlation in Fig. 3A and 3B looks to be driven by the low activity variants. That being said, the disruption of P1ex was previously shown to increase self-splicing in E. coli, but that was also based on the most active variants that survived selection. I would be interested in seeing an analysis of only sequences with fitness >-1.5-ish in terms of base composition and stability. Even though this comment is lengthy, I put this as a minor comment because it is neither novel nor controversial that the P1ex requires an intermediate stability. But, I think it is important not to over interpret differences in low activity data.

P8. The claim that noise from lowly abundant genotypes contains latent information is not well justified. Please add a qualifying statement – may or might – and discuss any important caveats.
